# Alternative surgical management of oroantral fistula using auricular cartilage

**DOI:** 10.4317/jced.51742

**Published:** 2015-04-01

**Authors:** Aydin Ozkan, Can-Engin Durmaz

**Affiliations:** 1 DDS, PhD, Oral and Maxillofacial Surgeon, Head of Surgical Infirmary, Land Forces Practice Center, Ankara, Turkey; 2 DDS, PhD, Oral and Maxillofacial Surgeon, Mevki Military Hospital, Department of Oral and Maxillofacial Surgery, Ankara, Turkey

## Abstract

One of the clinical complications encountered by oral and maxillofacial surgeons is oroantral communication (OAC) with subsequent formation of oroantral fistula (OAF). Many techniques and treatment modalities have been described for the management of OAC and OAF. There are advantages and disadvantages of all these techniques. We report a 21-year-old male patient who was admitted to our department for the presence of an OAF and was treated using an auricular cartilage graft. This technique may be useful to treat OAF and to provide a solid alveolar bone site for subsequent pre-implant surgery.

** Key words:**Auricular cartilage, implant surgery, oroantral fistula.

## Introduction

Oroantral fistula (OAF) is an epithelialized communication between the oral cavity and the maxillary sinus which has its origin from extraction of upper molar as the most common etiologic factor (incidence between 0.31% and 4.7%), followed by cysts, tumors, trauma, osteonecrosis and dehiscence following implant failure in atrophied posterior maxilla (1,2).

Many of surgical techniques to close OAF have been reported in the literature, such as buccal flap, palatal flap, buccal fat pad and relate modifications (3). They are their own advantages and disadvantages depending on the cases and the size of the defects occurred. Most of them rely on mobilizing the tissue and advancing the resultants flap into defect (2).

If the OAF has been a large bone defect or recurrence, conventional techniques may not be adequate closure of OAF. In this case we present an alternative surgical technique for the closure of OAF using auricular cartilage.

## Case Report

**Figure 1:**
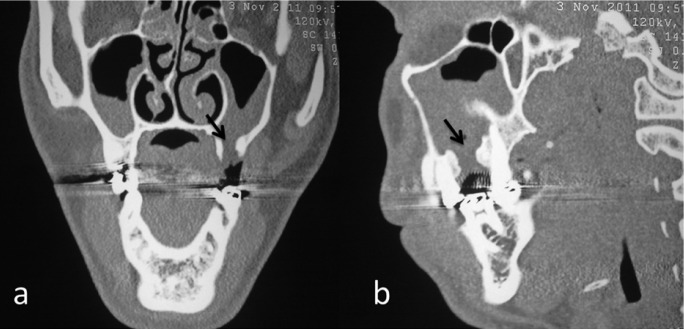
Pre-operative axial (a) and sagittal (b) scans showing OAF in the upper first molar region (black arrows).

A 21-year-old male patient admitted to our department with the complaint of nasal sporadic intraoral drainage since 2 years following traumatic extraction of upper left first molar tooth. There was no history of systemic disease. Intraoral examination revealed 2x3 mm mucosal opening in the region of left first molar tooth. Sagittal and axial computed tomography scans showed large destruction of bone without any evidence of foreign bodies (Fig. 1). Preoperative antimicrobial therapy was started to control the infection, after which surgery was scheduled.

**Figure 2:**
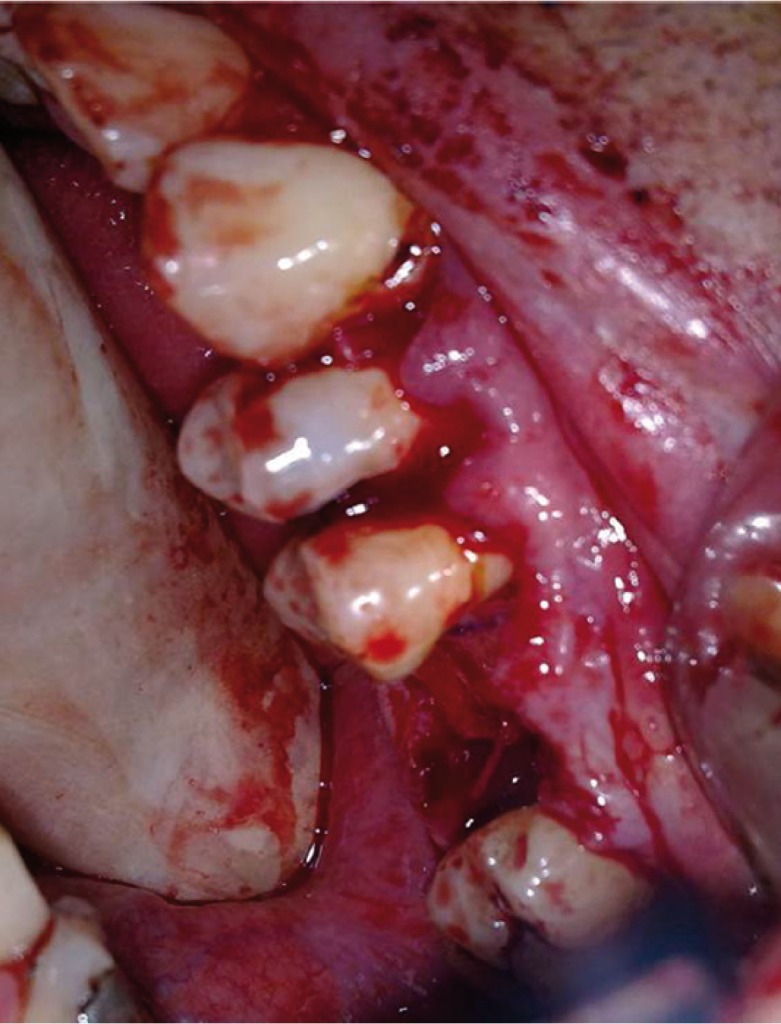
Pre-operative axial (a) and sagittal (b) scans showing OAF in the upper first molar region (black arrows).

Under general anesthesia, initially, epithelial lining of fistula was excised and granulation tissue was curetted. The intrasulcular horizontal incision with no vertical releasing incision was reflected from the left-side of canine to second molar. The horizontal incision began in the gingival suclus and was extended through the fibres of gingival attachment to the crestal bone (Fig. 2). After the bone destruction was exposure adequately, it was smoothed with the rotary instruments. The sinus was irrigated with normal saline solution.

**Figure 3:**
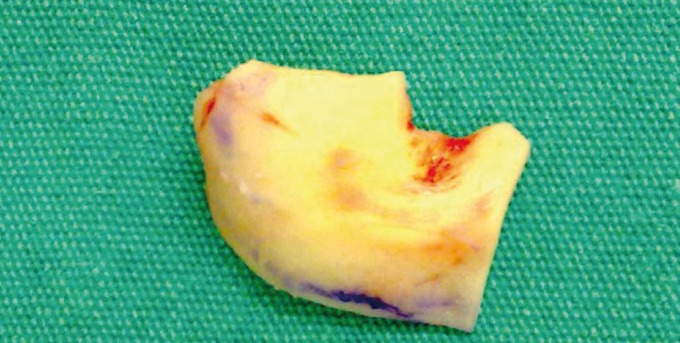
Photo showing harvested auricular cartilage.

After the preparation of the recipient site, an incision was made in the postauricular skin that overlies the emenitia of the concha. The skin and soft tissue were dissected and an auricular cartilage was sharply incised. Care is taken to preserve the cartilage of the antihelical fold as well as the crus helices. The anterior flap is elevated in the subperichondrial plane and the auricular cartilage is harvested (Fig. 3). Meticulous attention was given to hemostasis and the wound sutured by horizontal mattress technique with 6-0 nylon and a compressive dressing was applied.

**Figure 4:**
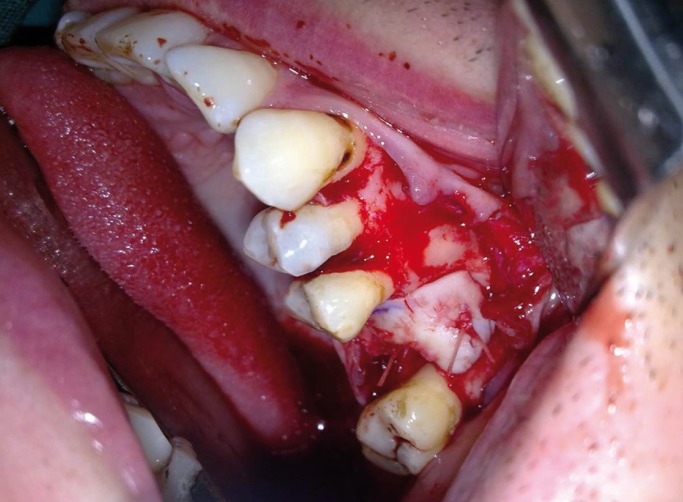
Intra-operative view showing stabilized auricular cartilage on the bone defect.

**Figure 5:**
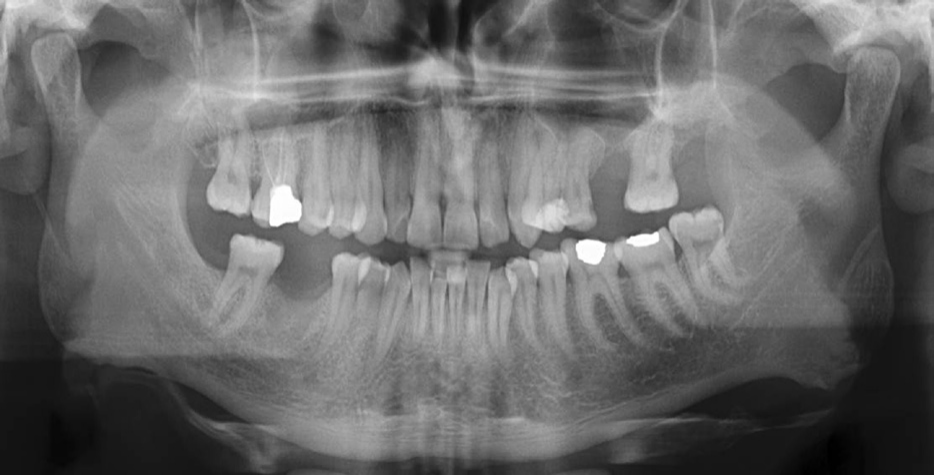
Six-month post-operative panoramic radiograph showing the defect filled new bone.

**Figure 6:**
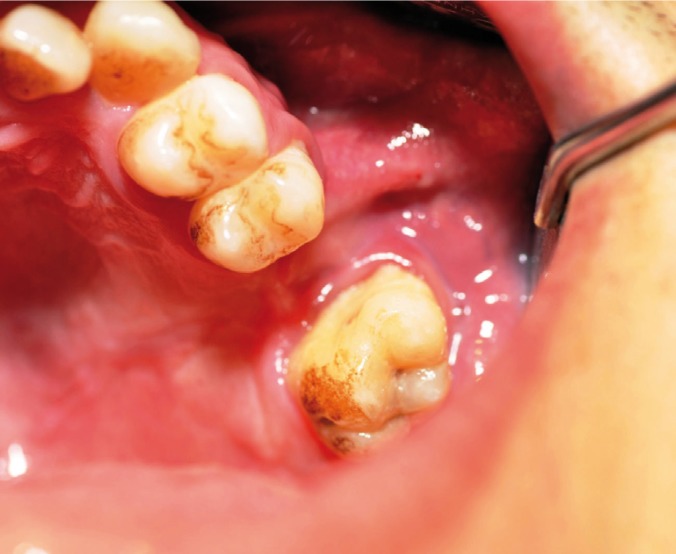
Intraoral post-operative appearance of the operation side after six months.

Harvested auricular cartilage graft was sutured over oroantral communication with 3/0 vicryl suture for stabilization (Fig. 4) and then the mucoperiosteal flap was sutured on the cartilage graft primarily with 3/0 silk suture. Routine postoperative instructions, including medications (antibiotics, analgesics and decongestant) and to avoid severe physical activities (nose blowing, sneezing, vigorous rinsing) that might raise the pressure within the para nasal sinuses are given for one week. Sutures were removed on the tenth day after operation and the postoperative course was uneventful. The patient was scheduled for regular follow up appointments. At the 6-month follow-up, bone destruction area was filled with new bone (Fig. 5) and the wound in the defect area become successfully epithelized without dehiscence (Fig. 6).

## Discussion

Different parameters including size and location of defect as well as its relationship to adjacent teeth, height of the alveolar ridge, persistence, presence of sinus disease and patient’s general health affect to choose the surgical technique for treatment of OAF (4). Vischer *et al.* (5) presented conventional methods especially buccal and palatal flap commonly used for closure of the OAF in the review of the literature. Our experiences show us that these methods are not sufficient for closure of OAF which has large bone defects and these techniques can be used single after surgical failure. In additionally, conventional techniques can reduce vestibular depth and cause lack of bone support (6,7).

Another major problem for the closure of large OAF is providing complete separation between the sinus membrane and the oral mucosa. As a result of that, a conjunction occurs between the Schneiderian membrane and mucosal tissue during the healing process and this complication makes difficult to perform implant rehabilitation and pre-implant surgical procedures such as sinus floor elevation (6-8). Therefore in the present study auricular cartilage graft is used as an alternative technique for closure of OAF.

Auricular cartilage graft can be manipulated easily, has the benefit of superior long term survival, is available for the head and neck region and is resistant to resorption and infection. Because of these reason it is commonly used in reconstructive surgery such as closure of palatal fistula and rhinoplasty (9). Isler *et al.* (10) used auricular cartilage for closure of OAF but they harvested the cartilage anterior approach and performed this for edentulous patient. Differently in our case the location of OAF was along the roots of neighboring teeth and we preferred a retroauricular incision. The posterior approach minimizes scar visibility and postoperative contour deformities. In additional if the OAF is near the teeth, solitary soft tissue closure may be concluded relapses.

Management of OAF is still a controversial topic. If the sinus is uninfected and communication is less than 3 mm in diameter healing will most likely spontaneously. If the communication fails to close spontaneously, it remains patent and epithelialized so that an OAF will develop (3). In this case, treatments of patients are so difficult and incidence of chronic sinusitis increases. In our opinion oroantral communication should be closed immediately in order to prevent sinusitis and the mucosal tissue and Schneiderian membrane should be separated by appropriate barriers.

In a conclusion, closure of the communications with cartilage graft substitutes is a valid alternative to flap based techniques. Conventional techniques cause matting of the mucosae and Schneiderian membrane so that elevation of the sinus membrane without disruption becomes impossible.
